# Genetic variation in *ST6GAL1* is a determinant of capecitabine and oxaliplatin induced hand‐foot syndrome

**DOI:** 10.1002/ijc.34046

**Published:** 2022-05-10

**Authors:** Katie Watts, Christopher Wills, Ayman Madi, Claire Palles, Timothy S. Maughan, Richard Kaplan, Nada A. Al‐Tassan, Rachel Kerr, David J. Kerr, Richard S. Houlston, Valentina Escott‐Price, Jeremy P. Cheadle

**Affiliations:** ^1^ Division of Cancer and Genetics, School of Medicine Cardiff University Cardiff UK; ^2^ The Clatterbridge Cancer Centre NHS Foundation Trust Wirral UK; ^3^ Institute of Cancer and Genomic Sciences, Institute of Biomedical Research University of Birmingham Birmingham UK; ^4^ CRUK/MRC Oxford Institute for Radiation Oncology University of Oxford Oxford UK; ^5^ MRC Clinical Trials Unit University College of London London UK; ^6^ Department of Genetics King Faisal Specialist Hospital and Research Center Riyadh Saudi Arabia; ^7^ Department of Oncology, Old Road Campus Research Building University of Oxford Oxford UK; ^8^ Nuffield Department of Clinical Laboratory Sciences University of Oxford, John Radcliffe Hospital Oxford UK; ^9^ Division of Genetics and Epidemiology The Institute of Cancer Research London UK; ^10^ Institute of Psychological Medicine and Clinical Neurosciences, School of Medicine Cardiff University Cardiff UK

**Keywords:** chemotherapy, colorectal cancer, genetics, *ST6GAL1*, toxicity, XELOX

## Abstract

Cancer patients treated with capecitabine and oxaliplatin (XELOX) often develop hand‐foot syndrome (HFS) or palmar‐plantar erythrodysesthesia. Genetic variation in *ST6GAL1* is a risk factor for type‐2 diabetes (T2D), a disease also associated with HFS. We analysed genome‐wide association data for 10 toxicities in advanced colorectal cancer (CRC) patients from the COIN and COIN‐B trials. One thousand and fifty‐five patients were treated with XELOX ± cetuximab and 745 with folinic acid, fluorouracil and oxaliplatin ± cetuximab. We also analysed rs6783836 in *ST6GAL1* with HFS in CRC patients from QUASAR2. Using UK Biobank data, we sought to confirm an association between *ST6GAL1* and T2D (17 384 cases, 317 887 controls) and analysed rs6783836 against markers of diabetes, inflammation and psoriasis. We found that 68% of patients from COIN and COIN‐B with grade 2‐3 HFS responded to treatment as compared to 58% with grade 0‐1 HFS (odds ratio [OR] = 1.1, 95% confidence interval [CI] = 1.02‐1.2, *P* = 2.0 × 10^−4^). HFS was also associated with improved overall survival (hazard ratio = 0.92, 95% CI = 0.84‐0.99, *P* = 4.6 × 10^−2^). rs6783836 at *ST6GAL1* was associated with HFS in patients treated with XELOX (OR = 3.1, 95% CI = 2.1‐4.6, *P =* 4.3 × 10^−8^) and was borderline significant in patients receiving capecitabine from QUASAR2, but with an opposite allele effect (OR = 0.66, 95% CI = 0.42‐1.03, *P* = .05). *ST6GAL1* was associated with T2D (lead SNP rs3887925, OR = 0.94, 95% CI = 0.92‐0.96, *P =* 1.2 × 10^−8^) and the rs6783836‐T allele was associated with lowered HbA1c levels (*P =* 5.9 × 10^−3^) and lymphocyte count (*P =* 2.7 × 10^−3^), and psoriasis (*P =* 7.5 × 10^−3^) beyond thresholds for multiple testing. In conclusion, HFS is a biomarker of treatment outcome and rs6783836 in *ST6GAL1* is a potential biomarker for HFS with links to T2D and inflammation.

## Graphical abstract

What's new?

Combination therapy with capecitabine and oxaliplatin (XELOX) in cancer patients can lead to hand‐foot syndrome (HFS), risk factors for which include older age, being female, and having pre‐existing conditions, such as diabetes and neuropathy. Here, investigation of HFS in XELOX‐treated patients with advanced colorectal cancer (CRC) shows that HFS toxicity is associated with improvements in treatment response and overall survival. In particular, the variant rs6783836 in *ST6GAL1*, a gene with roles in inflammation and type‐2 diabetes, was linked to HFS and glycated haemoglobin levels. The findings identify rs6783836 as a promising HFS biomarker in XELOX‐treated patients with advanced CRC.

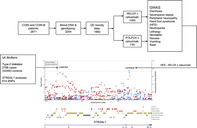

**Abbreviations**
CRC: colorectal cancer
CTCAE: Common Terminology Criteria for Adverse Events
eQTL: expression quantitative trait loci
GTEx: The Genotype‐Tissue Expression project database
GWAS: Genome‐Wide Association Study
QC: quality control
QUASAR2: QUick And Simple And Reliable trial
SNP: single nucleotide polymorphism

## 1 INTRODUCTION

Toxicity from chemotherapy may result in treatment discontinuation or dose reduction affecting the prospect of a cure in patients with cancer. Patients treated with capecitabine and oxaliplatin (XELOX) often develop hand‐foot syndrome (HFS), in which small amounts of the chemotherapeutic agent leaks out of capillaries into the hands and feet and damages the surrounding tissues.^1^ HFS has been suggested to be a biomarker of treatment efficacy with post hoc analyses from clinical trials of colorectal and breast cancer patients finding that grade 1+ HFS was associated with improved overall and progression‐free survival.^2^, ^3^ Established risk factors for HFS include being older, female, having pre‐existing peripheral neuropathy, circulation problems and diabetes.^4^, ^5^

Genetic variation in ST6 β‐galactoside α‐2,6‐sialyltransferase 1 (*ST6GAL1*) is associated with risk of developing type‐2 diabetes (T2D).^6^, ^7^ *ST6GAL1* catalyses the addition of α2,6‐linked sialic acids onto key surface glycoproteins. Increases in α2,6‐linked sialic acids have been linked to inflammatory conditions^8^ and *ST6GAL1* deficiency leads to increased inflammatory cell production,^9^ granulocyte recruitment^10^ and cytokine release.^11^ There is also substantial evidence that *ST6GAL1* plays an important role in cancer progression, and is overexpressed in numerous cancers including colorectal.^12^ High *ST6GAL1* expression has been associated with radioresistance and chemoresistance to several anticancer treatments, which ultimately leads to poorer patient outcomes.^13^, ^14^, ^15^, ^16^

Inherited genetic factors are being recognised to affect toxicity from chemotherapeutic agents; notably, rare variants in the dihydropyrimidine dehydrogenase gene are associated with 5‐fluorouracil toxicity. We have previously studied the relationship between common single nucleotide polymorphisms (SNPs) and 10 of the major toxicities, in patients with advanced colorectal cancer (CRC) from the COIN and COIN‐B^17^, ^18^, ^19^ clinical trials.^20^ Here, we extended our analysis and meta‐analysed those patients receiving XELOX ± cetuximab and, separately, folinic acid, fluorouracil and oxaliplatin (FOLFOX) ± cetuximab. We also sought to confirm an association between *ST6GAL1* and T2D, and understand their interrelationship with HFS by studying biomarkers of inflammation and psoriasis using data from the UK Biobank.

## 2 MATERIALS AND METHODS

### 2.1 Patients and samples

Two thousand six hundred and seventy‐one patients (mean age at randomisation of 62 years, range 18‐87, 36% female) with metastatic or locally advanced CRC recruited into the MRC clinical trials COIN (ISRCTN27286448)^17^, ^18^ and COIN‐B (ISRCTN3837568)^19^ were studied. None of the patients had previously received chemotherapy for advanced disease. COIN patients were randomised 1:1:1 to receive continuous oxaliplatin and fluoropyrimidine chemotherapy (n = 815), continuous chemotherapy with cetuximab (n = 815), or intermittent chemotherapy (n = 815). COIN‐B patients were randomised 1:1 to receive intermittent chemotherapy and cetuximab (n = 112) or intermittent chemotherapy and continuous cetuximab (n = 114). For the first 12‐weeks, treatments were identical in all patients apart from the choice of fluoropyrimidine (n = 1603, 60% received XELOX and n = 1068, 40% received FOLFOX) together with the randomisation of ±cetuximab (n = 1041, 39% received cetuximab). Blood DNA samples were prepared from 2244 of the 2671 patients.

### 2.2 Toxicities assessed

Assessment of toxicities was performed at 12 weeks, since at this point patients from all trial arms received identical levels of chemotherapy with or without cetuximab. This time point was also prior to any interruption to treatment for the intermittent therapy arms. Toxicities assessed were diarrhoea, neutropenic sepsis, peripheral neuropathy, HFS, neutropenia, lethargy, stomatitis, nausea, vomiting and rash graded by critical adverse events as per the Common Terminology Criteria for Adverse Events (CTCAE version 4.0) with the highest grade noted within the first 12 weeks of treatment. Patients with toxicities graded 2‐5 (G2‐5) were grouped and compared against those graded 0‐1 (G0‐1). For HFS, we compared patients with G2‐3 (G3 is the maximum) against those with G0‐1; we also considered a linear model of toxicity.

### 2.3 Patient outcome

Assessment of response was also performed at 12 weeks. Response was defined as complete or partial response using RECIST 1.0 guidelines and no response was defined as stable or progressive disease. Overall survival (OS) was defined as time from randomisation to death or date of last assessment.

### 2.4 Genotyping

Two thousand two hundred and forty‐four patient DNA samples were genotyped using Affymetrix Axiom Arrays according to the manufacturer's recommendations (Affymetrix, Santa Clara, California).^21^ After quality control (QC), SNP genotypes were available for 1950 patients.^21^ For 150 patients, no data on toxicity had been collected and these were excluded leaving 1800 for analysis. Prediction of untyped SNPs was carried out using IMPUTEv2 (v2.3.0) based on data from the 1000 Genomes Project as reference. We restricted our analysis to directly typed SNPs and imputed SNPs with INFO scores ≥0.8, a Hardy‐Weinberg equilibrium ≥1.0 × 10^−6^ and a minor allele frequency (MAF) ≥0.05.

### 2.5 Statistical analyses

We previously analysed 4 million SNPs for a relationship with each toxicity under univariate models in patients that received XELOX (n = 707), XELOX + cetuximab (n = 348), FOLFOX (n = 385) and FOLFOX + cetuximab (n = 360).^20^ Here, we incorporated covariates associated at *P* < .05 (Table S1) into the additive logistic models in Plink v1.9^22^ and meta‐analysed those patients receiving XELOX ± cetuximab (n = 1055) and, separately, FOLFOX ± cetuximab (n = 745). Meta‐analyses were run under a random effects model to account for the effect of cetuximab on toxicity, and results plotted in R studio using qqman.^23^ SNPs associated at genome‐wide significance (*P* < 5.0 × 10^−8^) were selected for further analyses. For survival analyses, cox proportional hazard regression models were used for both univariate and multivariate analyses. Results are reported in accordance with STREGA guidelines.^24^

MAGMA^25^ was used for gene and gene set analyses using data files from the NCBI 37.3 gene definitions and ~8500 predefined gene sets. Gene analyses were run under a SNP‐wise univariate model imposing a Bonferroni corrected significance threshold of *P =* 2.5 × 10^−6^. Gene set analyses were run under competitive models with a corrected significance threshold of *P =* 5.8 × 10^−6^.

Power to detect toxicity effect sizes was calculated using the genpwr package in R,^26^ based upon 70% power, *P =* 5.0 × 10^−8^ and SNPs with MAFs = 0.20; under these conditions we could identify SNPs with a mean OR of 2.8 (range 2‐4 dependent upon toxicity, Table 1).

**TABLE 1:** Patients with grade 2‐5 CTCAE toxicities at 12 weeks and detectable odds ratios at 70% power

	Frequency			
Toxicity	XELOX ± cetuximab	FOLFOX ± cetuximab	Detectable odds ratio	
	n (%)	n (%)	XELOX ± cetuximab	FOLFOX ± cetuximab
Diarrhoea	288 (27)	187 (25)	2	3
Neutropenic sepsis	6 (1)	63 (8)	NA	4
Peripheral neuropathy	154 (15)	73 (10)	2	3
Hand‐foot syndrome	109 (10)	65 (9)	3	4
Neutropenia	42 (4)	209 (28)	4	2
Lethargy	361 (34)	256 (34)	2	2
Stomatitis	61 (6)	150 (20)	4	3
Nausea	210 (20)	88 (12)	2	3
Vomiting	122 (12)	59 (8)	3	4
Rash	177 (17)	201 (27)	2	2

*Note*: Percentage of patients in parentheses. NA—for neutropenic sepsis in patients treated with XELOX ± cetuximab we had insufficient power to perform the genome‐wide association study. Patients with hand‐foot syndrome were graded 2‐3.

### 2.6 Independent replication

We attempted to replicate the association of rs6783836 with HFS using data from 927 patients with stage II or III CRCs enrolled in the Quick and Simple and Reliable trial (QUASAR2) comparing capecitabine or capecitabine plus bevacizumab.^27^ Patients were genotyped using the Illumina genome‐wide SNP panels (Human Hap 370, Human Hap 610 or Human Omni 2.5). Imputation was performed using IMPUTEv2 with 1000 genomes as reference. The INFO score for rs6783836 was 0.89. HFS was graded using the CTCAE scale and patients with G2‐3 (46%) were compared to those with G0‐1. Age was used as a covariate.

### 2.7 *ST6GAL1* variants and T2D

Six hundred and fourteen SNPs spanning *ST6GAL1* were tested for an association with T2D in UK Biobank participants under project application number 65833 (17 384 cases and 317 887 controls as of 1 January 2021). We restricted our analysis to directly typed SNPs and imputed SNPs with INFO scores ≥0.8, a Hardy‐Weinberg equilibrium ≥1.0 × 10^−6^ and a MAF ≥0.01. We also analysed the relationship between rs6783836 and diabetic skin lesions by logistic regression on 617 diabetic individuals with self‐reported open sores and 6605 diabetic controls (as of 1 July 2021).

### 2.8 Potential biomarkers of HFS

We analysed rs6783836 and potential biomarkers of HFS using participant data from the UK Biobank. We assessed seven markers of wound healing and/or inflammation: lymphocyte, neutrophil, monocyte, eosinophil, platelet and basophil counts (10^9^ cells/L) and C‐reactive protein levels (mg/L), and one marker for diabetes: glycated haemoglobin (HbA1c) levels (mmol/mol). Analyses were run using PHESANT.^28^ Lymphocyte count, HbA1c levels, platelet count, neutrophil count, c‐reactive protein levels and monocyte count were analysed under a linear regression and, basophil count and eosinophil count were analysed under an ordered logistic model as software default due to limited variation in the data. Results were held to a significance threshold of *P =* 6.3 × 10^−3^ (Bonferroni correction for eight tests, *P* = .05/8). We analysed rs6783836 as a potential regulator of inflammation by performing a univariate logistic regression on 4228 individuals from the UK Biobank with self‐reported psoriasis and 331 043 controls (as of 1 January 2021).

### 2.9 Additional bioinformatic analyses

The Genotype‐Tissue Expression (GTEx) project database was used to identify expression quantitative trait loci (eQTLs) for relevant SNPs (https://gtexportal.org/home). Fine‐mapping was used for SNPs at significant loci; conditional regression was first used to identify the number of causal variants and fine‐mapping was then run using PAINTOR.^29^ Credible sets of causal SNPs were assembled for 95% coverage.

## 3 RESULTS

### 3.1 Relationship between HFS and patient outcome

**FIGURE 1:**
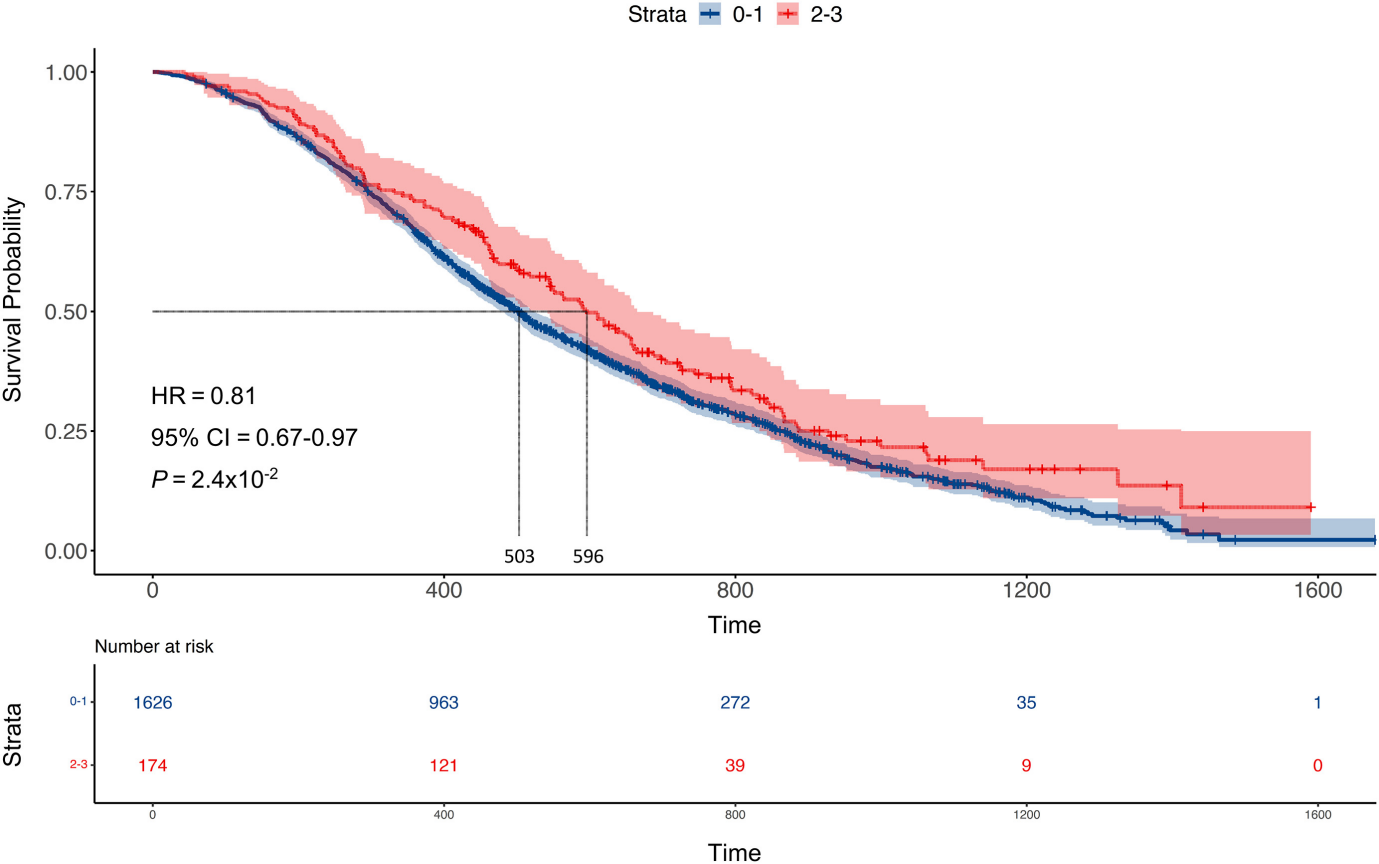
Kaplan‐Meier plot showing the relationship between hand‐foot syndrome (HFS) and overall survival (OS). The y‐axis represents survival probability and the x‐axis represents time (days). The blue line represents patients with G0‐1 HFS and the red line patients with G2‐3 HFS. Dotted lines show the median OS (596 days in those with G2‐3 HFS and 503 days in those with G0‐1 HFS). The *P*‐value was calculated using a cox proportional hazard regression [Color figure can be viewed at wileyonlinelibrary.com]

Overall, 174/1800 (10%) patients from COIN and COIN‐B developed G2‐3 HFS at 12 weeks (109/1055, 10% in the XELOX group and 65/745, 9% in the FOLFOX group, Table 1). HFS was predictive of treatment outcome (Table 2). 105/154 (68%) patients with G2‐3 HFS responded (had complete or partial response) to chemotherapy ± cetuximab at 12 weeks as compared to 831/1436 (58%) with G0‐1 HFS (odds ratio [OR] =1.6, 95% confidence intervals [CI] =1.1‐2.2, *P* = 1.4 × 10^−2^, univariate model). Under a multivariate model accounting for age, sex, disease site, World Health Organisation performance status, primary tumour resection status, white blood cell count, chemotherapy regimen and cetuximab status, this remained significant (OR = 1.1, 95% CI = 1.02‐1.2, *P* = 2.0 × 10^−2^). Median OS was 596 days in those with G2‐3 HFS and 503 days in those with G0‐1 HFS (hazard ratio [HR] = 0.81, 95% CI = 0.67‐0.97, *P* = 2.4 × 10^−2^, Figure 1); although, this did not remain significant under multivariate analysis (*P* = .15, Table 2). However, when HFS was assessed as a linear trait, the relationship with OS was significant under such analyses (G0 median survival = 499 days, G1 = 514 days, G2 = 596 days, G3 = 687 days, HR = 0.92, 95% CI = 0.84‐0.99, *P* = 4.6 × 10^−2^, Table 2). Cetuximab increased the frequency of HFS in patients treated with XELOX (56/348, 16% with and 53/707, 8% without cetuximab, *P* = 1.5 × 10^−5^) and FOLFOX (56/360, 16% with and 9/385, 2% without cetuximab, *P* = 2.7 × 10^−8^).

**TABLE 2:** Relationship between hand‐foot syndrome (HFS) and patient outcome in COIN and COIN‐B

Model	Grade of HFS (n)	Response at 12 weeks				Overall survival			
		% Responders	OR	95% CI	*P* (multivariate)	Median survival (days)	HR	95% CI	*P* (multivariate)
Grouped	0–1 (1626)	58	1.6	1.1–2.2	1.4 × 10^−2^ (2.0 × 10^−2^)	503	0.81	0.67–0.97	2.4 × 10^−2^ (0.15)
	2–3 (174)	68				596			
Linear	0 (1264)	56	1.3	1.2–1.6	1.4 × 10^−4^ (2.0 × 10^−4^)	499	0.90	0.83–0.97	5.8 × 10^−3^ (4.6 × 10^−2^)
	1 (362)	66				514			
	2 (144)	68				596			
	3 (30)	67				687			

*Note*: Response was defined as complete or partial response using RECIST 1.0 guidelines and no response was defined as stable or progressive disease. One thousand and eight hundred patients had data on overall survival and 1590 had data on response at 12 weeks. Covariates included in the multivariate analysis were age, sex, disease site, World Health Organisation performance status, primary tumour resection status, white blood cell count, chemotherapy regimen and cetuximab status.

### 3.2 Relationship between genetic variation at *ST6GAL1* and HFS

**FIGURE 2:**
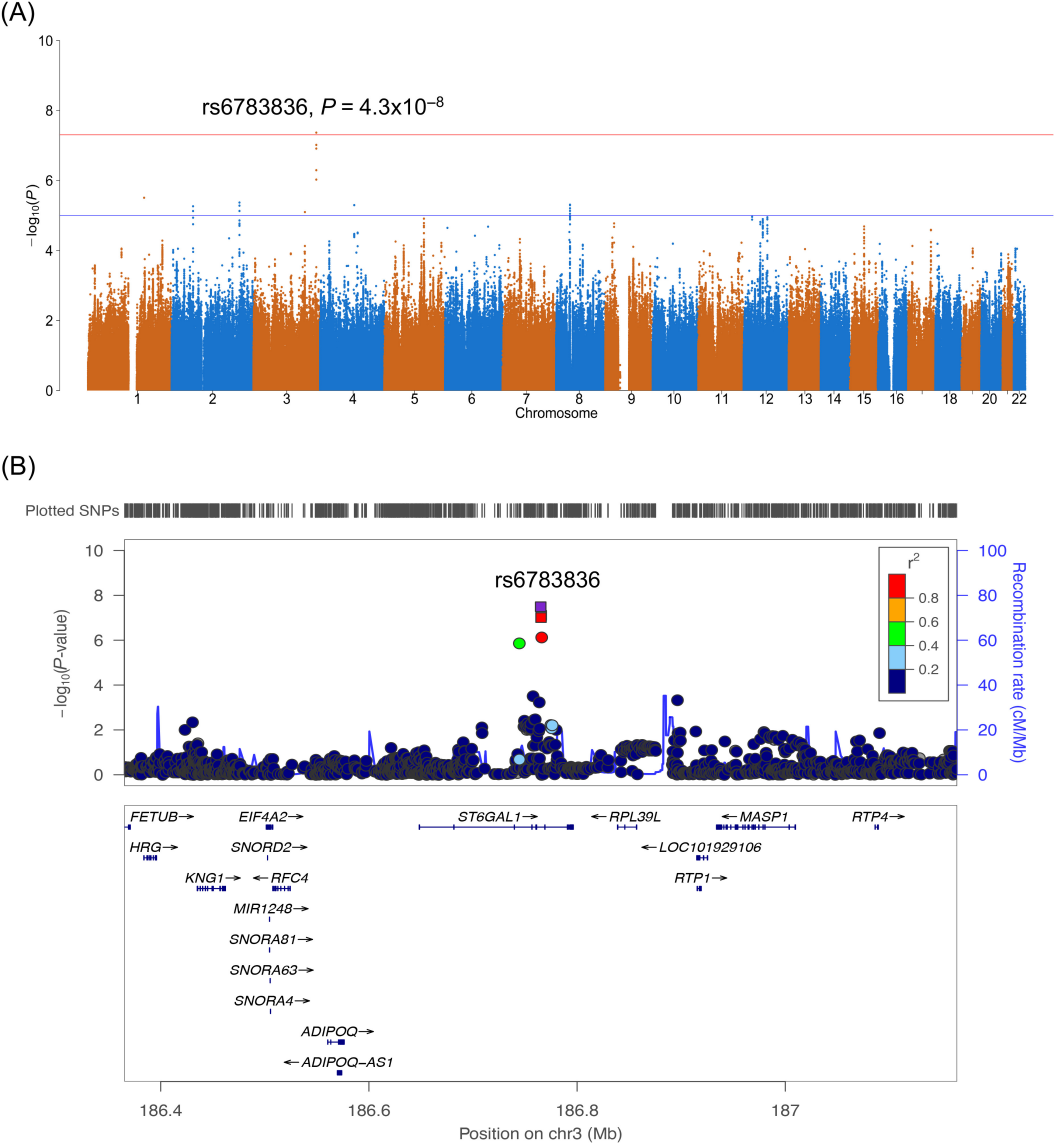
Regional plots for the association of rs6783836 with hand‐foot syndrome (HFS). (A) Manhattan plot of the association between single‐nucleotide polymorphism (SNP) genotype and HFS in patients treated with capecitabine and oxaliplatin. The red line corresponds to a *P =* 5.0 × 10^−8^ and the blue line *P =* 1.0 × 10^−5^. (B) Locus zoom plot shows results of the analysis for SNPs and recombination rates. −log_10_(*P*) (*y* axis) of the SNPs are shown according to their chromosomal positions (*x* axis). The sentinel SNP (purple) is labelled by its rsID. The colour intensity of each symbol reflects the extent of linkage disequilibrium with the sentinel SNP, deep blue (*r* ^2^ = 0) through to dark red (*r* ^2^ = 1.0). Genetic recombination rates, estimated using 1000 Genomes Project samples, are shown with a blue line. Physical positions are based on NCBI build 37 of the human genome. Also shown are the relative positions of genes and transcripts mapping to the region of association. Genes have been redrawn to show their relative positions; therefore, maps are not to physical scale. Fine‐mapping identified a credible set of 3 SNPs with rs6783836 having the highest posterior probability of 0.53 [Color figure can be viewed at wileyonlinelibrary.com]

**FIGURE 3:**
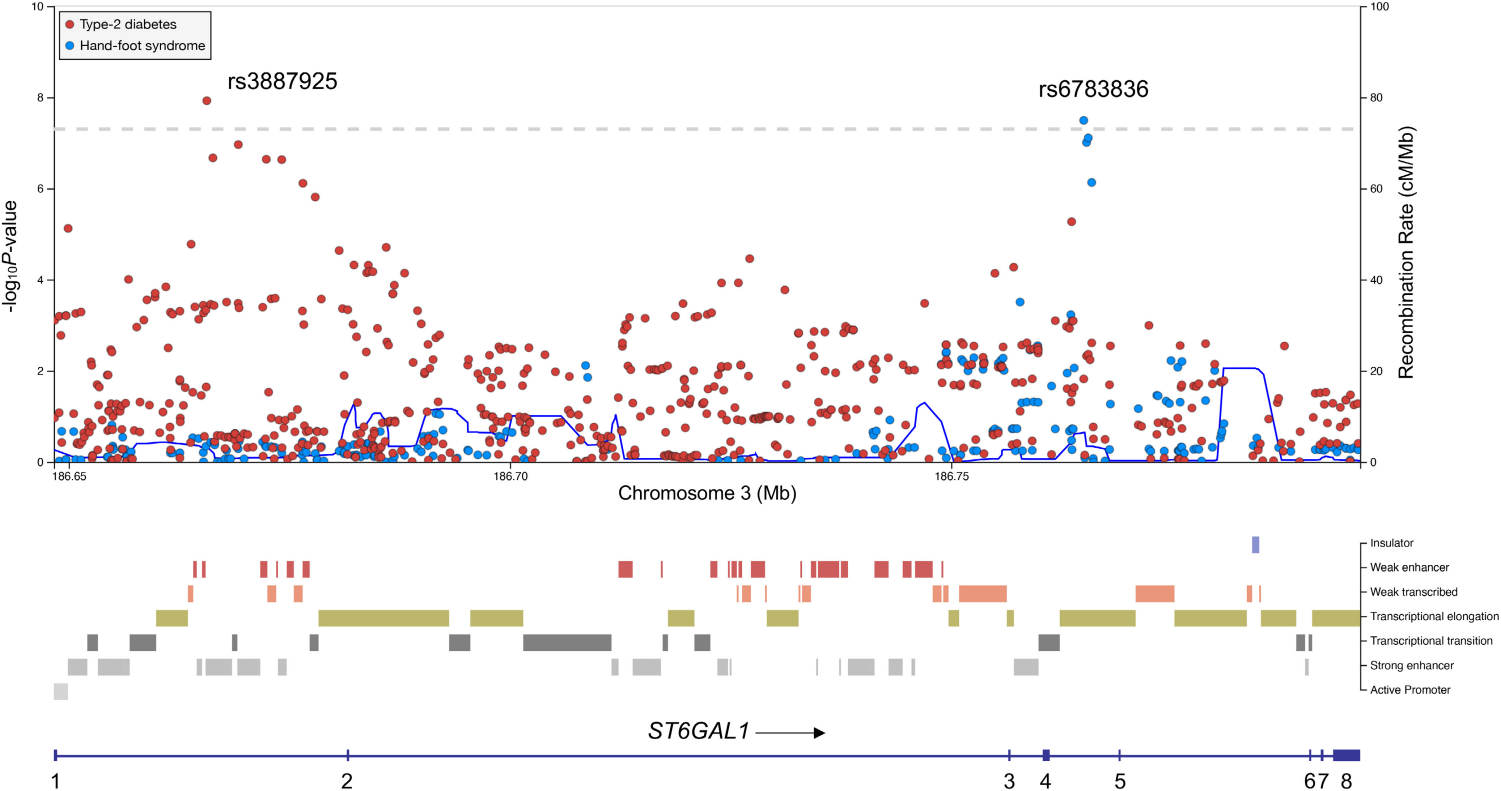
Layered locus zoom plot showing single‐nucleotide polymorphisms (SNPs) in *ST6GAL1* associated with hand‐foot syndrome (HFS) and type‐2 diabetes (T2D). Plot shows results of the analysis for SNPs and recombination rates. −log_10_(*P*) (*y* axis) of the SNPs are shown according to their chromosomal positions (*x* axis). The dashed line corresponds to a *P =* 5.0 × 10^−8^. Genetic recombination rates, estimated using 1000 Genomes Project samples, are shown with a blue line. Physical positions are based on NCBI build 37 of the human genome. Lead SNPs for HFS and T2D are indicated by their rsIDs. Also shown is the relative coding region of *ST6GAL1* and chromatin state annotations from ENCODE [Color figure can be viewed at wileyonlinelibrary.com]

rs6783836 at 3q27.3 was associated with HFS at genome‐wide significant levels in patients treated with XELOX (OR = 3.1, 95% CI = 2.1‐4.6, *P =* 4.3 × 10^−8^, Figure 2). Forty‐six percent (50/108) of patients with G2‐3 HFS carried rs6783836 in a heterozygous or homozygotes state for the minor allele as compared to 21% (200/934) of patients with G0‐1 HFS (Table 3). The association between rs6783836 and HFS was seen in patients treated with XELOX alone (OR = 3.3, 95% CI = 1.9‐5.7, *P* = 2.7 × 10^−5^) and in those treated with XELOX + cetuximab (OR = 2.9, 95% CI = 1.6‐5.1, *P* = 3.0 × 10^−4^, Table 3); cetuximab did not affect this relationship (*P*_interaction_ = 0.98). rs6783836 was not associated with HFS in patients treated with FOLFOX (OR = 0.86, 95% CI = 0.44‐1.7, *P =* .65) and the difference between regimens was significant (*P*_interaction_ = 1.0 × 10^−3^). rs6783836 was not associated with patient outcome regardless of chemotherapy regime (XELOX ± cetuximab, response OR = 1.0, 95% CI = 0.78‐1.4, *P* = .82 and OS HR = 0.95, 95% CI = 0.82‐1.1, *P* = .46; FOLFOX ± cetuximab, response OR = 0.77, 95% CI = 0.54‐1.1, *P* = .15 and OS HR = 1.0, 95% CI = 0.86‐1.2, *P* = .78). rs6783836 maps to intron 4 of *ST6GAL1* in a region involved in transcriptional elongation (Figure 3) and was not an eQTL.

**TABLE 3:** Relationship between rs6783836 and hand‐foot syndrome (HFS) in patients from COIN and COIN‐B treated with XELOX ± cetuximab

Treatment groups analysed	Total patients	Patients G0‐1 HFS			Patients G2‐3 HFS			OR	95% CI	*P*‐value
		wild type	heterozygous	homozygous	wild type	heterozygous	homozygous			
Meta‐analysis	1042	734	190	10	58	48	2	3.1	2.1–4.6	4.3 × 10^−8^
*Subgroups*:										
XELOX	699	520	121	5	30	21	2	3.3	1.9–5.7	2.7 × 10^−5^
XELOX + cetuximab	343	214	69	5	28	27	0	2.9	1.6–5.1	3.0 × 10^−4^

Abbreviations: CI, confidence intervals; OR, odds ratio; T, reference allele.

### 3.3 Investigating the relationship between rs6783836 and HFS in an independent cohort

rs6783836 was borderline significant for HFS in patients treated with capecitabine from QUASAR2 (OR = 0.66, 95% CI = 0.42‐1.03, *P* = .05) but with an opposite direction of effect to that found in COIN and COIN‐B (Table S2).

### 3.4 Investigating other variants, genes and pathways associated with toxicities

No other SNPs were associated with toxicities in COIN and COIN‐B at genome‐wide significant levels, but eight SNPs were suggestive of association (*P* < 1.0 × 10^−6^, Table S3). No genes were associated with toxicities after correction for multiple testing (data not shown). Four gene sets—peripheral neuropathy with response to food, neutropenia with dendritic spine development, diarrhoea with co‐receptor activity and skin rash with blood vessel endothelial cell migration, were associated after correction for multiple testing (Table S4).

### 3.5 Understanding the interrelationship between genetic variation in *ST6GAL1* , T2D and HFS

**FIGURE 4:**
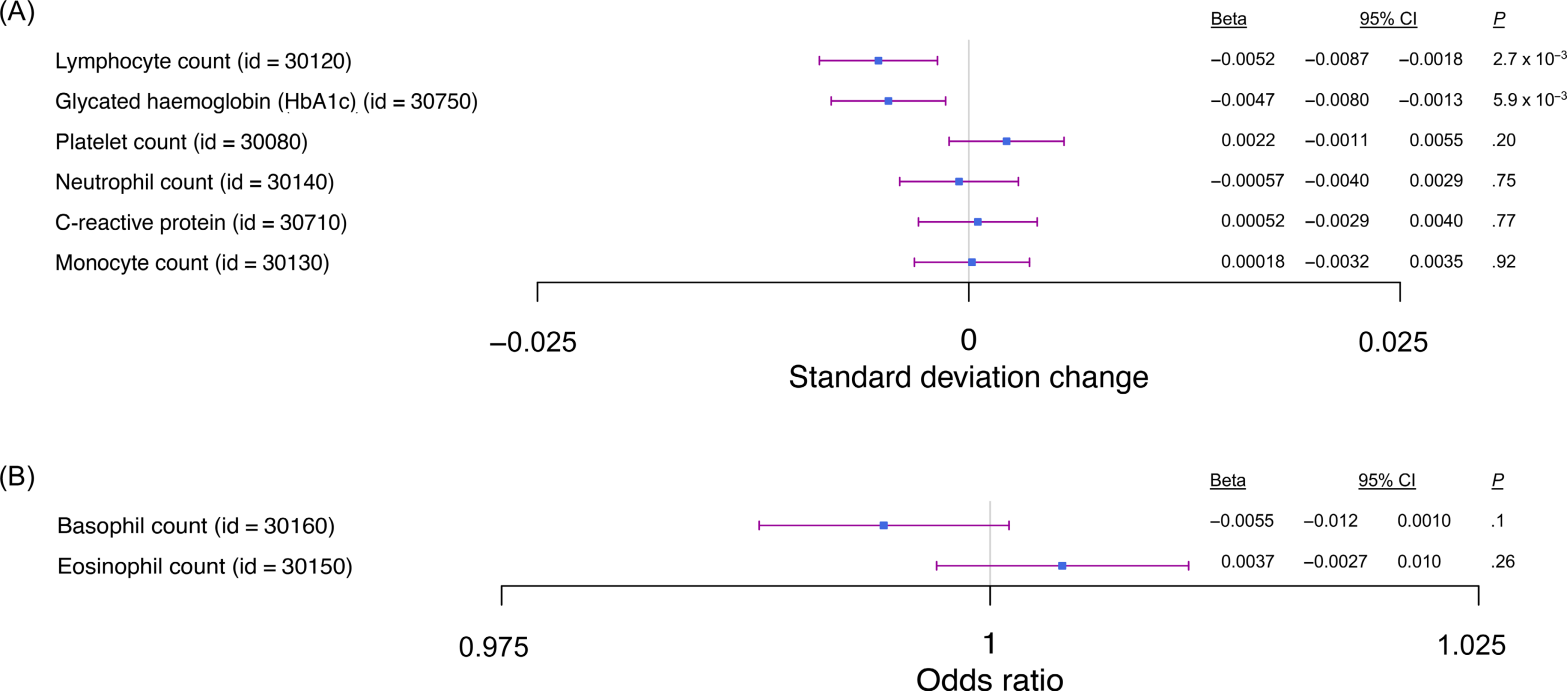
Relationship between rs6783836 and (A) continuous and (B) ordinal phenotypes. The *x* axis shows phenotype and respective UK Biobank ID, and the *y* axis shows SD change or odds ratio. Only lymphocyte count and glycated haemoglobin (HbA1c) were significantly associated with rs6783836 after Bonferroni correction for eight tests (*P* < 6.3 × 10^−3^) [Color figure can be viewed at wileyonlinelibrary.com]

rs3887925 in intron 1 of *ST6GAL1* was the lead SNP associated with T2D (OR = 0.94, 95% CI = 0.92‐0.96, *P =* 1.2 × 10^−8^, Figure 3), although rs6783836 was not associated with T2D (OR = 0.93, 95% CI = 0.85‐1.0, *P* = .07) nor diabetic skin lesions (OR = 1.1, 95% CI = 0.89‐1.3, *P* = .44). rs3887925 and rs6783836 were not in linkage disequilibrium (LD) (*D*′ = 0.26, *R* ^2^ = .01). The rs6783836‐T allele was associated with lowered lymphocyte count (beta = −0.0052, 95% CI = −0.0087 to −0.0018, *P =* 2.7 × 10^−3^) and lowered HbA1c levels (beta = −0.0047, 95% CI = −0.0080 to −0.0013, *P =* 5.9 × 10^−3^, Figure 4) that withstood correction for multiple testing. rs6783836 was also associated with psoriasis (OR = 0.91, 95% CI = 0.85‐0.98, *P =* 7.5 × 10^−3^).

## 4 DISCUSSION

It has previously been suggested that HFS may be a biomarker of efficacy to chemotherapy.^2^, ^3^ However, others have suggested that since HFS is a cumulative toxicity, there may be a bias for those living longer simply having more HFS due to having more treatment. Our data only considered HFS after 12 weeks of treatment and we found that patients with HFS had better response to chemotherapy at 12 weeks. We also observed an improvement in OS when analysed under a linear model of toxicity. Similarly, an exploratory analysis of two German trials noted an association between HFS and OS, and found no difference between patients with early and late HFS.^30^ Together, these data suggest that HFS should be tolerated where possible and that an understanding of the underlying mechanism may help improve treatment efficacy.

With regards to the underlying mechanism, we identified rs6783836 in *ST6GAL1* as a genome‐wide significant biomarker for HFS in patients treated with XELOX, with or without, cetuximab. Diabetics are at an increased risk of developing HFS and we confirmed an association for *ST6GAL1* with T2D, and also found that rs6783836 was associated with glycated haemoglobin levels, a marker routinely used in the diagnosis and monitoring of diabetes, supporting an interrelationship. *ST6GAL1* has a known role in inflammation^31^ and we found an association between rs6783836 and lymphocyte count and psoriasis. Given that others have associated *ST6GAL1* with psoriasis in an Asian population,^32^ these data support a link between these syndromes and an underlying defect in the inflammatory pathway. Further studies are necessary to understand whether rs6783836 activates or inactivates ST6GAL1; however, it is interesting to note that *ST6GAL1* knockout mice are susceptible to ionising radiation and exhibit weight loss, gastrointestinal permeability and diarrhoea.^33^

The odds ratios and betas for rs6783836 / *ST6GAL1* with T2D, lymphocyte count and psoriasis were in the opposite direction to HFS. Lin et al^34^ proposed a flip‐flop mechanism for allelic heterogeneity caused by interacting loci in weak LD and this has gained support from recent studies^35^, ^36^, ^37^, ^38^, ^39^ and may help explain our observations. Interestingly, the association with HFS was not found in patients treated with FOLFOX and was borderline significant, but with allele flipping, in patients from QUASAR2 treated with capecitabine alone. Further studies are therefore warranted to understand the underlying process and its specificity to particular therapeutic combinations.

We noted an association between peripheral neuropathy and genes involved in response to food which is supported by a previous observation linking diet to chemotherapy‐induced peripheral neuropathy (CIMP).^40^ Other forms of peripheral neuropathy have also been linked with diet.^41^, ^42^ Adopting vegetarianism has been shown to relieve symptoms in patients with diabetic neuropathy,^43^, ^44^ and there is evidence that taking multivitamins reduces the likelihood of a patient experiencing CIMP.^45^ Our data adds weight to this promising avenue for treatment of this toxicity.

## AUTHOR CONTRIBUTIONS

The work reported in the article has been performed by the authors, unless specified in the text. Jeremy P. Cheadle obtained funding for our study. The study was designed and directed by Jeremy P. Cheadle. Timothy S. Maughan was CI of COIN and Ayman Madi was a COIN trial fellow—both provided clinical advice and supported the translational research. Richard Kaplan managed the COIN and COIN‐B trials and facilitated access to the clinical data. Nada A. Al‐Tassan oversaw the genotyping and Richard S. Houlston oversaw the imputation and quality control. Claire Palles, Rachel Kerr and David J. Kerr provided data from QUASAR2. Christopher Wills oversaw quality control of the UK Biobank data. Katie Watts undertook all of the statistical analyses with supervision from Valentina Escott‐Price and Jeremy P. Cheadle. Katie Watts and Jeremy P. Cheadle interpreted the data with input from Christopher Wills and Valentina Escott‐Price. Katie Watts wrote the first draft of the article with subsequent input from Jeremy P. Cheadle and all authors provided comments.

## CONFLICT OF INTEREST

Timothy S. Maughan consults for AstraZeneca and receives personal fees from Pierre Fabre (IDMC services). Timothy S. Maughan received research funding from Merck KgAa and AstraZeneca. The institution where Timothy S. Maughan works receives funding from Bayer. David J. Kerr is a director of Oxford Cancer Biomarkers. All other authors have declared no conflicts of interest.

## ETHICS STATEMENT

All patients gave fully informed consent for bowel cancer research (approved by REC [04/MRE06/60]).

## Supporting information


**Appendix S1** Supporting Information.

## Data Availability

The GWAS summary statistics are available through the NHGRI‐EBI GWAS Catalogue under study accession numbers GCST90095054‐GCST9009572. Further details and other data that supports the findings of our study are available from the corresponding author upon request. This research was also conducted using the UK Biobank Resource under application number 65833.
