# Supplementary material for: Genetic variation in ST6GAL1 is a determinant of capecitabine and oxaliplatin induced hand‐foot syndrome
Source: Int J Cancer. 2022 May 10;151(6):957–66. doi: 10.1002/ijc.34046 (PMC9545609; doi:10.1002/ijc.34046)
Supplement: Supplementary file 1 — Appendix S1 Supporting Information. [file IJC-151-957-s001.pdf]

SUPPLEMENTARY INFORMATION

Genetic variation in *ST6GAL1* is a determinant of capecitabine and oxaliplatin induced hand-foot syndrome

Katie Watts, Christopher Wills, Ayman Madi, Claire Palles, Timothy S. Maughan, Richard Kaplan, Nada A. Al-Tassan, Rachel Kerr, David Kerr, Richard S. Houlston, Valentina Escott-Price and Jeremy P. Cheadle

Table of Contents

Supplementary Table S1. Covariates used in the genome-wide association studies **2**

Supplementary Table S2. Relationship between rs6783836 and hand-foot syndrome (HFS) in patients from QUASAR2 treated with capecitabine ± bevacizumab **3**

Supplementary Table S3. Single nucleotide polymorphisms (SNPs) associated with toxicities at P<1.0x10^{-6} **4**

Supplementary Table S4. MAGMA gene set analyses **5**

Supplementary Table S1. Covariates used in the genome-wide association studies

	Sex	Creatinine clearance	Location of primary tumour	Age	Platelet count	WHO performance status
Diarrhoea	**$3.3x10^{-2}$**	**$6.5x10^{-4}$**	0.18	0.65	0.86	**$3.8x10^{-2}$**
Neutropenic sepsis	**$4.6x10^{-4}$**	0.53	0.07	0.15	0.12	0.07
Peripheral neuropathy	**$3.1x10^{-2}$**	0.71	0.53	0.70	**$1.0x10^{-2}$**	0.39
Hand-foot syndrome	0.09	**$4.6x10^{-2}$**	0.46	**$1.9x10^{-3}$**	**$1.8x10^{-2}$**	0.82
Neutropenia	**$2.5x10^{-4}$**	**$1.9x10^{-2}$**	0.51	0.20	**$2.9x10^{-3}$**	0.13
Lethargy	**$3.9x10^{-3}$**	0.21	**$3.6x10^{-2}$**	0.18	0.74	**$1.4x10^{-5}$**
Stomatitis	**$4.5x10^{-3}$**	0.76	0.98	0.06	0.07	**$3.0x10^{-3}$**
Nausea	**$5.3x10^{-4}$**	**$8.3x10^{-3}$**	**$8.5x10^{-3}$**	**$1.1x10^{-2}$**	0.08	**$3.0x10^{-2}$**
Vomiting	0.09	**$3.5x10^{-2}$**	**$2.6x10^{-2}$**	**$2.0x10^{-2}$**	0.78	0.79
Rash	**$1.3x10^{-3}$**	0.50	0.09	**$1.8x10^{-2}$**	0.07	**$1.1x10^{-2}$**

Logistic model also had chemotherapy regimen and cetuximab as terms added. Covariates associated at $P<0.05$ (in bold) were included in the genome-wide association studies.

Supplementary Table S2. Relationship between rs6783836 and hand-foot syndrome (HFS) in patients from QUASAR2 treated with capecitabine ± bevacizumab

Treatment groups analysed	Total patients	Patients G0-1 HFS		Patients G2-3 HFS		OR	95% CI	*P*-value
		without rs6783836	with rs6783836	without rs6783836	with rs6783836			
Capecitabine	441	171	51	184	35	0.7	0.4-1.0	0.05
Capecitabine + bevacizumab	487	164	38	220	65	1.3	0.9-1.9	0.24
Meta-analysis	928	335	89	404	100	0.9	0.7-1.3	0.71

Reference allele = T, OR = Odds ratio, CI = Confidence intervals. Patients with rs6783836 included heterozygous carriers and homozygotes for the minor allele.

Supplementary Table S3. Single nucleotide polymorphisms (SNPs) associated with toxicities at *P*<1.0x10^{-6}

Treatment	Toxicity	Lead SNP	Cytoband	OR	95% CI	*P*-value
**XELOX ± cetuximab**	Hand-foot syndrome	rs6783836	3q27.3	3.1	2.1-4.6	4.3x10^{-8}
	Stomatitis	rs12433034	14q11.2	2.8	1.9-4.3	4.8x10^{-7}
		rs143685874	4q34.1	3.5	2.1-5.8	9.2x10^{-7}
	Diarrhoea	rs11295266	2q21.2	1.8	1.4-2.2	6.9x10^{-7}
**FOLFOX ± cetuximab**	Stomatitis	rs12029003	1p13.2	3.0	1.9-4.5	3.7x10^{-7}
		rs141748690	5q23.1	3.7	2.2-6.2	5.0x10^{-7}
		rs1233378	6p22.1	2.4	1.7-3.4	9.6x10^{-7}
	Neutropenia	rs825249	16q23.3	2.2	1.6-3.0	3.0x10^{-7}
	Nausea	rs79848933	15q22.2	3.7	2.2-6.2	5.3x10^{-7}

Supplementary Table S4. MAGMA gene set analyses

Treatment	Toxicity	Pathway	*P*-value
**XELOX ± cetuximab**	Peripheral neuropathy	Response to food	3.0×10^{-7}
	Neutropenia	Negative regulation of dendritic spine development	5.5×10^{-6}
**FOLFOX ± cetuximab**	Diarrhoea	Coreceptor activity	2.8×10^{-7}
	Rash	Negative regulation of blood vessel endothelial cell migration	2.7×10^{-6}

Genome-wide significance was a Bonferroni corrected significance threshold of $P<5.6 \times 10^{-6}$.
